# Optimization of Microwave-Assisted Extraction of Polyphenols from *Crataegus monogyna* L.

**DOI:** 10.3390/antiox14030357

**Published:** 2025-03-18

**Authors:** Adina I. Gavrila, Emilia J. Damian, Anca Rosca, Ioan Calinescu, Camelia Hodosan, Ioana Popa

**Affiliations:** 1 Faculty of Chemical Engineering and Biotechnologies, National University of Science and Technology Politehnica Bucharest, 011061 Bucharest, Romania; adina.gavrila@upb.ro (A.I.G.); anca_maria.rosca@stud.fim.upb.ro (A.R.); ioan.calinescu@upb.ro (I.C.); 2 Research & Development Department, Teva Pharmaceuticals S.R.L., 011171 Bucharest, Romania; emiliajulia.deliu@tevapharm.com; 3 Faculty of Engineering and Animal Production, University of Agronomic Sciences and Veterinary Medicine, 011464 Bucharest, Romania

**Keywords:** hawthorn, bioactive compounds, microwave, antioxidant activity, factorial design

## Abstract

Hawthorns (*Crataegus monogyna* L.) contain numerous bioactive compounds, with its extracts demonstrating health benefits. This study focused on optimizing a more sustainable extraction method, specifically microwave-assisted extraction (MAE), to obtain polyphenols from hawthorn leaves and flowers. HPLC/UV analysis identified key compounds, including gallic and chlorogenic acids, isoquercetin, rutin, hyperoside, vitexin, and quercetin. Principal component analysis (PCA) assessed correlations between extraction conditions, total phenolic content (TPC), and key compounds. PCA grouped conditions into three clusters, with two remaining ungrouped. The highest vitexin, rutin, and isoquercetin contents were achieved at 60 °C for 10 min using 160–500 μm particles in 75% ethanol (20/1 ratio) or 50% ethanol (20/1 and 30/1 ratios). An ungrouped condition (60 °C, 10 min, < 160 μm particles, 50% ethanol, 20/1 ratio) produced a higher TPC and greater gallic acid, chlorogenic acid, and hyperoside concentrations. The TPC and antioxidant activity (AA) of the extracts were optimized using a 2^3^ full factorial design, with temperature, ethanol concentration, and solvent-to-plant ratio as variables. Optimal MAE conditions (S/P_opt_ = 20.4 mL/g, T_opt_ = 65 °C, and EtOH_opt_ = 60%) yielded a TPC of 116.23 ± 2.85 mg GAE/g DM and an AA of 237.6 ± 6.33 mg TE/g DM using hawthorn leaves and flowers. Summarizing the above, to maximize phytocompound content, a one-factor-at-a-time design identified extraction parameters, but its limitations led to a 2^3^ full factorial design. The latter effectively optimized the TPC and AA, while PCA revealed correlations between extraction parameters, total phenolics, and key compounds.

## 1. Introduction

In recent years, consumers have tended towards natural and eco-friendly products related to pharmaceuticals, food, cosmetology, and agronomy. In this context, the processing of plant materials shows promise due to their richness in phytocompounds that enhance human health and promote overall well-being. Among the bioactive compounds found in plants, those with neuroprotective effects have recently garnered significant interest. Nowadays, a significant number of people suffer from neurodegenerative diseases, which poses a critical public health issue [1]. It is generally acknowledged that the development and progression of neurodegenerative diseases are boosted by oxidative stress and inflammation [2], leading to damage in cellular and mitochondrial function, as well as dysfunction of deoxyribonucleic acid repair systems [3]. A higher intake of antioxidants can delay the onset of these disorders and improve the quality of life for already diagnosed patients. Polyphenols, plant secondary metabolites known for their potent antioxidant capacity, are categorized into flavonoids and nonflavonoids based on their chemical structure [1]. Polyphenols primarily exert neuroprotective effects by eliminating reactive oxygen species [4], crossing the blood–brain barrier [5], and chelating metal ions [6]. They also enhance neurotrophic factor concentrations and directly bind to their receptors [7], activating and modulating pathways that support neuronal growth [8], survival [9], proliferation [10], and plasticity [11], thereby enhancing memory, cognition, and learning [12]. Furthermore, polyphenol intake is generally free of adverse side effects.

Hawthorn (*Crataegus monogyna* L.) is a member of the genus *Crataegus*, one of the most intriguing genera within the Rosaceae family. The *Crataegus* genus is widely distributed across Europe, North America, temperate zones of Asia, and Africa. Hawthorn is a semi-deciduous shrub or small tree, typically with thorns, and generally grows to a height of 5–15 m [13]. It is rich in bioactive compounds, including polyphenols (phenolic acids and flavonoids), terpenoids, lignans, organic acids, nitrogenous compounds, and vitamins [14]. These phytoconstituents provide neuroprotective [15], antioxidant [16], anti-inflammatory and hepatoprotective [17], antibacterial [18], and immunomodulatory [19] properties to the extracts of hawthorn leaves.

The recovery of these bioactive compounds can be achieved through their extraction using both conventional and unconventional methods. The most widely used classical procedures are Soxhlet extraction and maceration [20]. However, these techniques involve extensive extraction times and high temperatures (in the case of the Soxhlet method), which consequently lead to a high energy consumption. Additionally, they require large volumes of solvent. These disadvantages make conventional methods less selective and efficient. Innovative techniques such as ultrasound-assisted [21], supercritical fluid [22], pressurized liquid [23], membranes [24], and microwave-assisted [25] extractions can overcome these drawbacks. Microwave-assisted extraction (MAE) is acknowledged as a more sustainable technique. This approach refers to an eco-friendly method for producing high-quality and safe extracts while minimizing time, solvent use, and energy consumption [26]. MAE involves using microwave energy to promote the movement of liquid molecules, facilitating the efficient extraction of bioactive compounds. It relies on the impact of electromagnetic radiation on a material capable of absorbing this energy and transforming it into heat. Consequently, microwave energy rapidly penetrates the plant matrix, increasing the internal temperature, causing overheating and subsequent vaporization. This phenomenon leads to the disruption of cell walls and the easier release of bioactive compounds [27].

High-Performance Liquid Chromatography (HPLC) stands out as the most effective method for analysing polyphenols in plant extracts and their derivatives. It is ideal for identifying and quantifying these bioactive compounds in complex matrices. Given the diverse chemical forms in which polyphenols can be found across different samples, precise analytical techniques are required to ensure accurate detection. This capability is essential for research in fields such as pharmacology, food science, and natural product chemistry, where polyphenols’ biological activities and health benefits are of great interest [28,29].

Although some research [20,25,30] focuses on MAE of polyphenols from hawthorn leaves and/or flowers, numerous trials are still required to determine the optimal parameters for maximizing the extraction yield of bioactive compounds. A one-factor-at-a-time experimental design has been a widely used technique for years to assess the impact of a broad range of parameter levels in an experiment. However, this method is not very useful in screening and evaluating the most significant factors and their interactions with each other. Design of Experiments is a useful tool for optimizing the extraction process, as it is a statistical method that enhances resource utilization and overall efficiency [31]. The existing literature reports only on the optimization of MAE of polyphenols from hawthorn fruits [32]. Hawthorn leaves and flowers have only been used in the optimization of supercritical fluid [22] and infusion [30] extraction methods for polyphenols. Thus, the novelty of the present study lies in the optimization of MAE of polyphenols from hawthorn leaves and flowers. Due to the limitations of the one-factor-at-a-time experimental design in evaluating factor significance and interactions, a two-level (2^3^) full factorial design was applied to maximize the bioactive compounds content obtained by MAE. This strategy effectively identifies the most influential factors and optimal conditions for enhancing polyphenols content and antioxidant activity (AA) in the extracts. Additionally, principal component analysis (PCA) was conducted to examine correlations between extraction parameters, total phenolic content (TPC), and key compounds, particularly those with neuroprotective potential.

## 2. Materials and Methods

### 2.1. Materials

Hawthorn leaves and flowers (*Crataegus monogyna* L.), marketed by Fares^®^ (Laboratoarele Fares Biovital SRL, Orăștie, Romania) in sealed bags of 50 g, were purchased from a local store (Bucharest, Romania). The experiment’s reproducibility was ensured by using hawthorn leaves and flowers from the same lot (19/2023). The humidity of the raw material was measured at 5.7% (w) using a moisture analyzer (PMB 202 Moisture Analyzer, Adam Equipment Co., Ltd., Bletchley, UK). Hawthorn leaves and flowers were processed by grinding and sieving to obtain three particle fractions with different sizes: smaller than 160 μm, 160–500 μm, and 500–1000 μm, respectively. The raw material was stored at 4–5° C until it was used for polyphenols extraction.

The standards for determining polyphenols and antioxidant activity were gallic acid and Trolox, respectively, purchased from Sigma-Aldrich Co, Bucharest, Romania. Ethanol (96%), copper chloride, neocuproine, ammonium acetate, Folin–Ciocalteu reagent, and sodium carbonate, also purchased from Sigma-Aldrich Co, Bucharest, Romania, were of analytical grade.

The standards used for HPLC analysis were gallic and chlorogenic acids, vitexin, rutin, hyperoside, isoquercetin, and quercetin purchased from Sigma-Aldrich Co, Bucharest, Romania.

### 2.2. Microwave-Assisted Extraction Procedure

The polyphenols were extracted from hawthorn leaves and flowers using MAE with the Biotage®Initiator microwave applicator (Biotage Sweden AB; Uppsala, Sweden). Hawthorn leaves and flowers of varying granulations (smaller than 160, 160–500, and 500–1000 μm) were accurately weighted and mixed with a solvent consisting of 0, 25, 50, 75, and 100% ethanol in water. The solvent-to-plant material ratio was varied as follows: 5, 10, 20, and 30 mL/g. A solvent volume of 20 mL was used for each experiment, and the plant material was weighed according to the solvent-to-plant material ratio for each extraction. The experiments were carried out at different temperatures (50, 60, and 70 °C) for 5, 10, 15, and 20 min. The microwave power varied from 15 to 35 W depending on the extraction time and temperature. All extractions were performed with a stirring rate of 900 rpm. In order to highlight the efficiency of MAE, a comparative experiment was conducted under the optimal extraction conditions achieved by MAE (ethanol concentration of 50%, extraction time of 10 min, temperature of 60 °C, solvent-to-plant ratio of 20 mL/g, stirring rate of 900 rpm, and particles size of 160–500 μm) using a conventional method. The latter involved using the same extraction vessel as the MAE, which was placed in a water bath, while the extraction mixture was heated with a temperature-controlled heating plate equipped with a magnetic stirrer. Finally, all extraction mixtures were separated by centrifugation at 5000 rpm and room temperature for 10 min. The extracts were freshly analysed in order to determine the TPC, AA, and polyphenol profile via HPLC. All experiments were performed in triplicate.

### 2.3. Total Phenolic Content Determination

The TPC of extracts was determined using the Folin–Ciocalteu assay, as described in our previous work [33]. The absorbance of the resulting solution was measured at 760 nm. The TPC is expressed as milligrams of gallic acid equivalents per gram of dry hawthorn leaves and flowers (mg GAE/g DM), based on a standard curve derived from a gallic acid solution with concentrations ranging from 0 to 5 mg/mL.

### 2.4. Antioxidant Activity Determination

The AA of hawthorn leaves and flowers extracts was assessed using the Cupric Reducing Antioxidant Capacity method, as described in our previous work [33]. The absorbance of the resulting solution was measured at 450 nm. The AA is expressed as milligrams of Trolox equivalents per gram of dry hawthorn leaves and flowers (mg TE/g DM), based on a standard curve derived from a Trolox solution with concentrations ranging from 0 to 0.25 mg/mL.

### 2.5. High-Performance Liquid Chromatography Analysis

The resulting extracts were further subjected to HPLC analysis in order to evaluate the polyphenols profile. The analyses were performed with a Jasco apparatus (ABL&E-JASCO România S.R.L., Cluj-Napoca, Romania) equipped with a UV-2075 detector, PU-2080 plus pump, LG-2080_4 gradient unit, DG-2080_4 degasser, and a Teknokroma Nucleosil 100 C18 (10 μm, 250 × 0.4 mm ID) separation column. The method used was gradient elution, and the separation was performed at room temperature. The analyses were conducted at a flow rate of 0.5 mL/min using water with 2% acetic acid (*v*/*v*) as solvent A and methanol as solvent B. The analytes were detected at a wavelength of 270 nm. Identification was performed by comparing retention times with known standards, while polyphenols quantification was based on peak area height. To eliminate potential interferences from other compounds present in the extracts, the polyphenols were re-extracted using diethyl ether as the solvent, following a procedure described in our previous work [34]. Quantification was carried out using calibration curves prepared with varying concentrations of polyphenol standards in 50% aqueous ethanol, adjusted to match the concentrations found in the extracts. As a result, the calibration curve covered the following concentration ranges: 50–150 mg/L for gallic acid, 50–150 mg/L for chlorogenic acid, 80–200 mg/L for vitexin, 70–200 mg/L for rutin, 50–200 mg/L for hyperoside, and 50–150 mg/L for quercetin. This specific dilution range was chosen to achieve optimal linearity, with good correlation coefficients ranging from 0.9834 to 0.9918.

### 2.6. Statistical Analysis

Triplicate measurements (n = 3) for TPC and AA determinations were carried out, with the data reported as the mean value ± standard deviation. The variations in the data were evaluated using a univariate one-way ANOVA using XLSTAT Version 2019.1 (Addinsoft, New York, NY, USA), as described in our previous work [33]. A confidence level of 95% was employed in the statistical analysis. PCA was used to explain the variations between extraction conditions and TPC and the main components from hawthorn extracts.

### 2.7. Experimental Setup and Factorial Analysis

A two-level factorial analysis was performed on three variables, namely solvent-to-plant ratio (mL/g), extraction temperature (°C), and ethanol concentration (%). In 12 experimental runs, each variable was randomized and evaluated at two coded levels, and two responses were recorded: TPC (mg GAE/g DM) and AA (mg TE/g DM). The significance of the model in the experiment and *p*-value was determined using the analysis of variance (ANOVA). The experiment was designed using Design Expert software® (Version 23.1.6, Stat-Ease 360, Inc., Minneapolis, MN, USA).

## 3. Results and Discussion

Polyphenols have been thoroughly investigated in recent decades for their potential to prevent and treat age-related neurodegenerative diseases. Due to the survival, differentiation, and enhancement of neuronal function and regeneration, diets that contain polyphenols have been demonstrated to offer advantages for preserving cognitive abilities [35]. Moreover, polyphenols stop the advancement of neurodegenerative diseases by enhancing cognition, memory, and learning [1].

Polyphenols, including the flavonoid subclass, are the main constituents of hawthorn, like hyperoside in fruits and flowers and vitexin in leaves. They may have a significant role in the protection of chronic degenerative illnesses through a fruit and vegetable diet, particularly in the fight against atherosclerosis and cancer. The basis for the biological effect rationalization of a plant therefore lies in the identification and quantification of the key phenolic chemicals accumulating in the plant, which are often derived from their antiradical, antioxidant, and anti-inflammatory activities [36].

The main polyphenols identified in hawthorn extracts by comparing the retention times of the commercial standards under the same conditions were gallic acid (5.945 min), chlorogenic acid (20.447 min), vitexin (25.382 min), rutin (32.889 min), hyperoside (34.948 min), and quercetin (62.265 min). Three polyphenols identified in the hawthorn leaves and flowers extracts have a potential neuroprotective effect. Vitexin reduces the loss of brain cells seen in neurodegenerative illnesses and acute shocks by upregulating cell survival pathways and downregulating proinflammatory and apoptotic signalling pathways [37]. Hyperoside, often referred to as quercetin-3-O-β-D-galactopyranoside, is a flavonol glycoside molecule with numerous biological activities, such as anti-inflammatory, antidepressant, antioxidant, vascular protector, and neuroprotection [38]. As one of the most powerful antioxidants derived from plants, quercetin is a major flavonoid that is more frequently present in edible plants. The neuroprotective effects of quercetin have been documented in a number of investigations, utilizing both in vitro and in vivo models of neurodegenerative illnesses, including ischemia, traumatic damage, cognitive impairment, and Parkinson’s and Huntington’s diseases [39].

### 3.1. Conventional Extraction vs. Microwave-Assisted Extraction

MAE is a current sustainable technique for the extraction of bioactive compounds from plant materials, being an important alternative in the extraction process due to its advantages, such as reduction of extraction time, selectivity, and control of the heating process [27]. To assess the effect of MAE on the TPC, a comparative analysis between microwave and conventional extraction methods was performed. Conventional extraction techniques are based on a process of permeation and solubilization of the intracellular components from plant material to the extract, which makes the difference from the MAE method. The entire volume of the plant material sample is heated from the inside using microwave heating. This contrasts with traditional heating, which requires contact with a heated surface in order to conduct heat and receives its heat from the outside. Furthermore, the water inside the plant cells is heated using microwave radiation in situ [27].

The influence of the heating type (conventional and MAE) on the TPC and the chemical composition of the polyphenolic extracts determined by HPLC analysis, as well as their concentration are presented in Table 1.

A higher content of polyphenols was observed with MAE. Results indicated a significant difference between conventional and MAE (*p* < 0.05). The highest extraction yield of individual bioactive compounds in the extract was achieved by MAE compared to conventional methods for compounds with a higher retention time, as shown in Table 1.

Owing to the principle of microwaves, which involves breaking the plant matrix and permitting solvent penetration, the accessibility of the solvent to the vegetal matrix should be enhanced. Using conventional techniques, bioactive compounds extraction is a laborious solvent-based procedure that relies on heat and mixing to speed up the mass transfer rate in the extraction system. On the other hand, it has been stated that MAE uses molecule interaction with the electromagnetic field to produce fast mass transfer to the extraction solvent. The plant cell wall can also be disrupted by microwave energy, which releases polyphenols into the solvent more quickly. On the other hand, the temperature rise and treatment duration are directly correlated with higher microwave power [40]. The outcomes showed that MAE can extract the targeted compounds from the hawthorn leaves and flowers without degrading them. Zivanovic et al. evaluated the phenolic profile of hawthorn leaves extracts obtained by macerating the leaves in 70% ethanol overnight, followed by a second maceration of the plant residues [41]. The results showed that hyperoside (13.6 mg/g), quercetin (0.1 mg/g), and rutin (2.3 mg/g) were present in concentrations comparable to those in the present study. However, vitexin (0.09 mg/g) and gallic acid (0.003 mg/g) were found in much lower concentrations. This difference could be attributed to the significantly longer extraction time used in their study compared with the conventional or MAE methods employed in the present work, which could lead to the degradation of some bioactive compounds. Regarding TPC, Zivanovic et al. reported a maximum value of 190 mg GAE/g DM [41]. The higher content, compared to the present study, could be due to the much longer extraction time, which may be beneficial for some polyphenolic compounds. However, Żurek et al. reported similar results to the present study for a 30 min ultrasound-assisted extraction: approximately 112 mg GAE/g DM, for both hawthorn leaves (60 mg/g) and flowers (52 mg/g) [16].

### 3.2. Influence of Time on the Extraction Efficiency of Bioactive Compounds

The extraction duration is a critical component that needs to be regulated to prevent the risk of heat deterioration and oxidation, which can affect the extract’s composition. The quantity of extracted bioactive phytochemicals rises over time until equilibrium is achieved between the compounds dissolved by the solvent and those remaining unextracted in the plant cells. The effect of extraction time can be observed through the comparative analysis of experimental data obtained from polyphenol extraction using microwave heating at different extraction times. The chemical composition and TPC of hawthorn polyphenol extracts obtained at these varying extraction times are depicted in Table 2.

Generally, increasing the extraction time leads to a higher amount of extracted polyphenols, but there is a risk of their degradation. As shown in Table 2, there are no significant differences after 10 min, as the presence of microwaves shortens the extraction time. As noted in Table 2, Folin–Ciocalteu results align with HPLC findings, as concentrations after 10 min of extraction are comparable to those at longer times. Ngoc et al. extracted polyphenols using a microwave method for 10 and 30 min, obtaining quite similar results for both extraction times. This suggests that, in terms of TPC, 10 min of extraction is sufficient [30].

### 3.3. Influence of Particle Size on the Extraction Efficiency of Bioactive Compounds

A crucial factor affecting extraction is the plant particle size. Decreasing the vegetal material dimension by grinding, the cell walls break down and the diffusivity of the bioactive substances increases. To enhance the contact surface between sample and solvent, the size of plant material should be small. When the size of the plant material is reduced, it will give the solvent a higher surface area to penetrate, produce a rupture of the cell, and improve mass transfer, all of which will lead to a successful extraction. The shape of the vegetal material is very important because, after being suspended in the solvent, its previously uneven shape becomes smooth and round. The solvent then creates a stagnant layer around the plant material, which could hinder the transfer of the secondary metabolites because they need to diffuse through this stationary layer [42].

Since the raw material consisted of a mixture of flowers and dried hawthorn leaves of different sizes, the plant material was divided into different fractions in order to assess the influence of granulation on the extraction efficiency of the bioactive compounds. Thus, three fractions of plant material were used: smaller than 160 µm, 160–500 µm, and 500–1000 µm. Table 3 illustrates the effect of sample particle size on MAE yield and the content of major compounds. TPC analysis shows an increase in total polyphenolic concentration as granulation decreases, with a significant difference between the 500–1000 µm and 160 µm fractions (*p* < 0.05).

Reducing the particle size led to an increase in broken cells, which aided in the mobilization of active components. Overall, as the sample particle size decreased, the overall polyphenolic content increased due to the enhanced contact surface area of the plant with the ethanol solvent used for extraction. Also, the smallest plant size led to the extraction of a higher amount of chlorogenic acid, hyperoside, and quercetin. The highest amount of vitexin and rutin was obtained for the 160–500 µm fraction. In contrast, gallic acid exhibited a different behavior, with higher extraction yields observed in larger particle sizes. This may be attributed to the greater stability and preservation of gallic acid in larger particles, as it is sensitive to environmental conditions such as light, heat, and oxygen. Smaller particles, while increasing surface area, expose gallic acid to harsher conditions, potentially leading to its degradation. Larger particles, on the other hand, provide a more stable environment, protecting gallic acid from degradation and ensuring higher recovery during extraction [43]. Samples with too tiny particle size complicate the extraction process and may require extra clean-up procedures, even with the improvement in MAE yields. Since the highest amount of bioactive compounds was achieved for particle dimension lower than 500 µm, this fraction was used for the following experiments. Ngoc et al. performed a MAE on raw and 100 µm-ground hawthorn flowers. The study showed a significant increase in TPC with a decrease in particle size of the plant material (23 and 35 mg GAE/g DM for raw and 100 µm-ground hawthorn flowers, respectively) [30]. The higher TPC achieved in the present study is likely due to the use of a mixture of leaves and flowers as plant material, rather than flowers alone as in the study by Ngoc et al.

### 3.4. Influence of Temperature on the Extraction Efficiency of Bioactive Compounds

Temperature is an important factor in the solid–liquid extraction process. To break down the plant material matrix in a way that allows secondary metabolites to diffuse and dissolve into the solvent, sufficient microwave power is required to generate the necessary heat for this process. Increasing microwave power can reduce extraction time and enhance yield [44]. On the other hand, applying excessive power without cooling the extraction mixture may lower the yield due to the degradation of heat-sensitive compounds. Generally, the extraction yield increases with higher microwave power until it reaches a point where the intensification becomes negligible or begins to drop. Microwave power plays a key role by creating localized heating within the sample, acting as the driving force in MAE to degrade the plant matrix, enabling bioactive compounds to diffuse into the solvent [45]. Microwave power and extraction temperature are interconnected. Without external cooling of the extraction mixture, if microwave power increases, so does the extraction temperature. Increasing the temperature enhances the solubility and boosts the solvent extraction effectiveness by reducing its viscosity and surface tension [27].

TPC analysis showed no significant differences at higher temperatures, though the concentration at 60 °C was higher than at the other two temperatures (Table 4).

However, the results from the HPLC analyses revealed differences in the quality of the extracts. At 50 and 70 °C, gallic acid and chlorogenic acid were predominant compared to the sample heated at 60 °C. The statistical analysis (ANOVA, Duncan test) indicated that only gallic and chlorogenic acids were significantly higher at 50 °C, while all the other components were better recovered at 60 °C. Raising the temperature accelerates the movement of polyphenols and solvent molecules through hydrogen bonding, thereby enhancing the exudation and diffusion of polyphenols more quickly. This process facilitates the release and distribution of polyphenols within plant materials. However, when the temperature exceeds 60 °C, the extraction rate of total polyphenols begins to drop, as excessive heat can lead to thermal degradation of polyphenols. High temperatures may cause dehydration, dehydroxylation, and other chemical bond disruptions that damage the molecular structure of polyphenols [46]. As a result, 60 °C was chosen for further experimentation.

### 3.5. Influence of Ethanol Concentration on the Extraction Efficiency of Bioactive Compounds

A key factor in the success of MAE is the careful selection of the solvent. The solvent’s dielectric constant, or that of a solvent mixture, is particularly important for ensuring an efficient and selective extraction process [27]. In MAE, microwaves selectively heat the sample, promoting the release of target compounds.

Materials with higher dielectric constants are better able to absorb microwave energy and convert it into heat, making extraction more efficient [42]. The dielectric constant defines the capacity of a material to absorb microwave energy and generate heat, which enhances the extraction of desired compounds during MAE. Therefore, choosing the right solvent is crucial and must be correlated with the chemical characteristics of the target compounds. Polar solvents such as ethanol, methanol, and ethyl acetate are commonly used to extract hydrophilic components from plants. When selecting a solvent for extraction, several factors must be considered, including its solubility for the target compounds, boiling point, reactivity, viscosity, recovery rate, vapor pressure, safety, toxicity, and cost. In some cases, it is beneficial to use aqueous solutions of organic solvents, as the presence of water improves the solvent penetration into the plant material, resulting in more effective heating [44]. Ethanol is widely used as a solvent, not only because it is a good absorber of microwave energy, but also because it effectively extracts a wide range of secondary metabolites. Additionally, the growing demand for eco-friendly extraction methods has led to the use of less toxic polar biosolvents like ethanol. Despite its potential hazards, such as flammability and explosiveness, ethanol remains popular due to its affordability, biodegradability, and availability [47].

The influence of different ethanol concentrations in water—25%, 50% and 75% (*v*/*v*)—on the extraction yield of polyphenols from hawthorn flowers and leaves was studied. The TPC and the main components of the extracts are depicted in Table 5.

As shown in Table 5, the highest concentration of polyphenolic compounds was achieved with 50% ethanol. This result can be explained by the fact that at lower ethanol concentrations, organic compounds are not extracted, while higher concentrations hinder the dissolution of water-soluble compounds. The solubility of polyphenols in water is determined by their polarity. Therefore, the choice of the solvent-to-water ratio depends on the polyphenol composition. Statistical analysis (ANOVA, Duncan test) pointed out that rutin, hyperoside, and quercetin were better recovered with 75% ethanol concentration in water, vitexin and chlorogenic acid with 50% aqueous ethanol, while the highest isoquercetin amount was achieved for a more aqueous solution (25% ethanol in water). The organic solvent (ethanol) dissolves the cell membranes and less polar polyphenols, while water dissolves the more polar polyphenols. Water also causes the plant material to swell, allowing the solvent to penetrate the solid matrix more easily, thus increasing the extractability of polyphenols [42]. Martino et al. identified vitexin and hyperoside in hawthorn leaf extracts obtained via microwave-assisted solvent extraction using HPLC [20]. The results showed that both polyphenols are present in higher concentrations when 50% ethanol was used as the solvent, compared with 96% ethanol (approximately 4.4 and 2.2 mg/g for 50% and 96% ethanol, respectively, for hyperoside, and 1.2 and 0.5 mg/g for vitexin) [20]. The differences in concentrations compared to the present study may be attributed to varying growth conditions, leaf maturity, etc., and to the shorter extraction time (3 min) in the study by Martino et al.

### 3.6. Influence of Solvent-to-Plant Ratio on the Extraction Efficiency of Bioactive Compounds

The solvent-to-plant ratio is an important parameter because it affects the amount of heat required for the extraction process. A higher solvent volume can affect the heating process generated by microwaves, as the solvent tends to absorb the microwaves. Excessive absorption by the solvent can prevent a sufficient amount of microwaves from passing through and into the plant matrix above the solvent layer. As a result, the plant material may not be heated uniformly, which is crucial for breaking down the cell wall and releasing the desired compounds [48]. On the other hand, using a low amount of solvent can hinder the movement of secondary metabolites within the plant matrix, creating a barrier to mass transfer. An appropriate amount of solvent is required to fully destroy the plant material during microwave irradiation. However, increasing the solvent-to-material ratio does not necessarily enhance extraction efficiency, as it can lead to uneven distribution and insufficient microwave exposure [49]. The solvent volume must always be sufficient to immerse the entire sample during extraction.

To examine the influence of the solvent-to-plant ratio on the TPC and extracts chemical composition, different ratios (5, 10, 20, and 30 mL/g) were tested. As shown in Table 6, TPC increased significantly (*p* < 0.05) at a solvent-to-plant ratio of 10 mL/g, with no significant rise thereafter. This behavior could be attributed to excessive absorption of microwave energy by the solvent at larger volumes during microwave application, reducing the available energy for cell disruption, which is necessary for effective extraction of the targeted analyte [48].

### 3.7. Principal Component Analysis

A PCA biplot was performed to evaluate the correlations between the extraction conditions, TPC, and the main components from hawthorn extracts. Two primary components emerged from the multivariate analysis of the data, accounting for 74.36% of the variability. The results were assessed using the sum of the first two main components. Of the overall variability, the first component contribution is 54.60%, while for the second component 29.76%.

Two eigenvalues higher than 1 corresponding to PC1 (3.57) and PC2 (2.38) are presented in the PCA outcomes. In Table 7 the variable coordinates (factor loadings) on the factor-plane PC1–PC2 are presented with the significant levels bolded.

**Figure 1:**
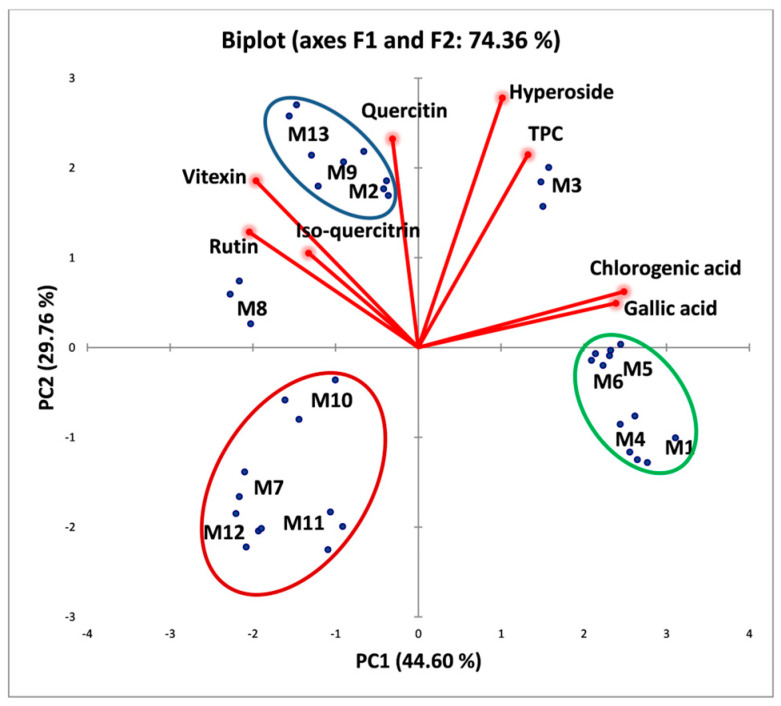
Principal component analysis (PCA) of bioactive components of hawthorn extracts and their extraction conditions (M1–M13). (Methods: M1—Conventional, 60 °C, S/P = 20 mL/g, <500 µm, 50% EtOH; M2—MW, 60 °C, S/P = 20 mL/g, <500 µm, 50% EtOH; M3—MW, 60 °C, S/P = 20 mL/g, <160 µm, 50% EtOH; M4—MW, 60 °C, S/P = 20 mL/g, >500 µm, 50% EtOH; M5—MW, 50 °C, S/P = 20 mL/g, <5 00 µm, 50% EtOH; M6—MW, 70 °C, S/P = 20 mL/g, <500 µm, 50% EtOH; M7—MW, 60 °C, S/P = 20 mL/g, <500 µm, 0% EtOH; M8—MW, 60 °C, S/P = 20 mL/g, <500 µm, 25% EtOH; M9—MW, 60 °C, S/P = 20 mL/g, <500 µm, 75% EtOH; M10—MW, 60 °C, S/P = 20 mL/g, <500 µm, 100% EtOH; M11—MW, 60 °C, S/P = 5 mL/g, <500 µm, 50% EtOH; M12—MW, 60 °C, S/P = 10 mL/g, <500 µm, 50% EtOH; M13—MW, 60 °C, S/P = 30 mL/g, <500 µm, 50% EtOH).

The loading plot shown in Figure 1 explains the correlations between the components and the variables. The closer variables show the comparable chemical composition of the extracts. On the other hand, a sample that is far apart from the others may have compositional features that are significantly different. The PCA graph also shows the difference between samples within individual species. By analyzing the distribution of data points in relation to the variables, three distinct groups corresponding to different processing methods were distinguished (groups highlighted by red, blue, and green ellipses).

PC1 was positively correlated with gallic acid and chlorogenic acid, and inversely correlated with vitexin and rutin. PC2 had a positive correlation with quercetin, hyperoside, and TPC (Table 7).

The PCA biplot presented in Figure 1 and correlation matrix shown in Table 8 indicate several key characteristics. Vitexin is directly correlated with rutin and isoquercetin (0.523 ≤ r ≤ 0.874), while quercetin shows a correlation with vitexin, rutin, and hyperoside (0.326 ≤ r ≤ 0.479). Additionally, hyperoside is positively correlated with gallic acid and chlorogenic acid (0.531 ≤ r ≤ 0.545). The group highlighted by the blue ellipse (samples M2, M9, and M13) has a higher content of vitexin, rutin, and isoquercetin compared to the group highlighted by the red ellipse (samples M7, M10, M11, and M12), discrimination on PC1. Furthermore, sample M3 contains higher amounts of hyperoside, gallic acid, and chlorogenic acid than the samples within the group highlighted by the green ellipse, also showing discrimination on PC1.

### 3.8. Design of Experiment 2^3^ Factorial Program

The optimal operating conditions for MAE are typically determined through statistical optimization studies. The screening of three independent variables (factors), namely solvent-to-plant ratio (S/P = 10–30 mL/g), temperature (t = 50–70 °C), and ethanol concentration (EtOH = 25–75%) was conducted using 2^3^ factorial design (Table 9 and Table 10). The effects of these process factors on dependent variables (responses), i.e., TPC and AA, were quantified using statistical models [31]. Experimental runs (1–8 in Table 9) were performed at 2 levels (minimum and maximum) of process factors.

As shown in Table 9, TPC values ranged from 56.42 to 107.9 mg GAE/g DM, while AA values ranged from 190.04 to 261.73 mg TE/g DM. As depicted in Table 10, both models had *p*-values < 0.0001, lack-of-fit *p*-values > 0.05 (0.1604 for TPC and 0.232 for AA), and F-values of 3.60 (TPC) and 2.57 (AA), indicating that the lack of fit is not significant relative to the pure error. There is a 16.04% (TPC) and 23.22% (AA) chance that the lack of fit F-values could occur due to the noise. For both responses, R^2^ was higher than 0.97 and adjusted coefficients (adj. R^2^) were higher than 0.95. The predicted R^2^ values (0.9160 for TPC and 0.9092 for AA) are in agreement with the adjusted R^2^ values (0.9746 for TPC and 0.9657 for AA), the differences being less than 0.2, outcomes confirmed by the low coefficient of variation (CV). These results indicated the good fitting accuracy of both models, and they might be used for the following prediction and correlation analysis.

#### 3.8.1. Effect of MAE Variables on the Polyphenol’s Extraction

**Figure 2:**
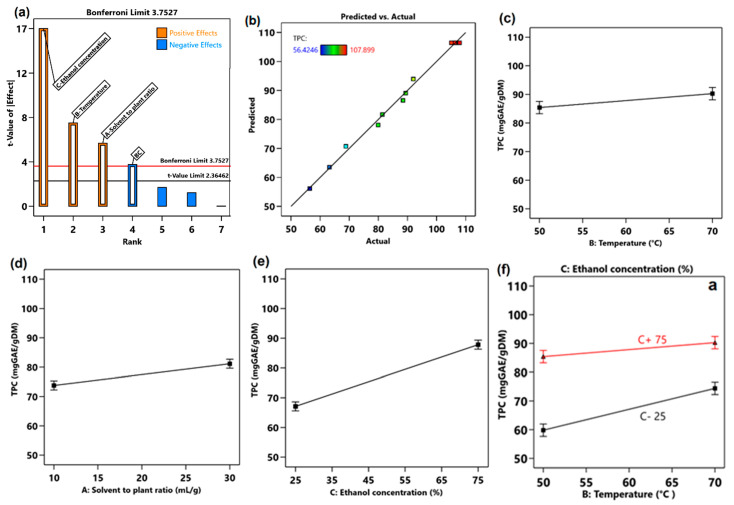
(**a**) Pareto chart for TPC, (**b**) actual vs. predicted result, (**c**) effect of temperature, (**d**) solvent-to-plant ratio, (**e**) ethanol concentration, and (**f**) interaction of B and C on TPC.

The effect of MAE factors on the TPC are shown in Figure 2. The experimental model provided by a two-level factorial design was used to generate the half-normal probability plot and Pareto chart (Figure 2a) to highlight the primary and interaction effects on TPC. The Pareto chart displays the absolute value of the effects from the largest to the smallest. For this reason, only the interaction between the two factors temperature and ethanol concentration (BC) was included. Consequently, the process response TPC can be predicted by a polynomial regression Equation (1) obtained after eliminating statistically non-significant terms.

Y = 77.46 + 3.68 × A + 4.86 × B + 10.36 × C − 2.43 × BC,(1)

where Y is the TPC of the hawthorn leaves and flowers extracts.

The Pareto chart provides a description of the interactions and effects of factors. Based on the Pareto chart of TPC (Figure 2a), the t-value limit is 2.36462 and below this value, any factor or interaction will not be significant., In Figure 2a, the Pareto chart showed that all factors and the interaction between ethanol concentration and temperature are significant, and the ethanol concentration was the most contributing factor in producing the highest TPC from hawthorn leaves and flowers. In contrast, all the interactions are non-significant because they fell below the t-value limit. A comparison of the predicted and actual outcomes is depicted in Figure 2b, and a good correlation between these results can be noticed, without a significant difference between the predicted and experimental values. As shown in Figure 2c–f and Table 10 (A), the effects of the extraction temperature, solvent-to-plant ratio, and ethanol concentration are significant (*p* < 0.05) and raising these parameters, lead to a higher TPC of the extracts. The contributing factors on the TPC of hawthorn extracts are in the next order: ethanol concentration (24.8%) > temperature (5.5%) > solvent-to-plant ratio (3.1%). Temperature and solvent-to-plant ratio show a less significant effect on TPC (*p* > 0.01). Moreover, the interaction between two factors (B and C) also contributed to the higher recovery of polyphenols, especially at lower ethanol concentration (Figure 2f).

#### 3.8.2. Effect of MAE Variables on the Antioxidant Activity of Extracts

**Figure 3:**
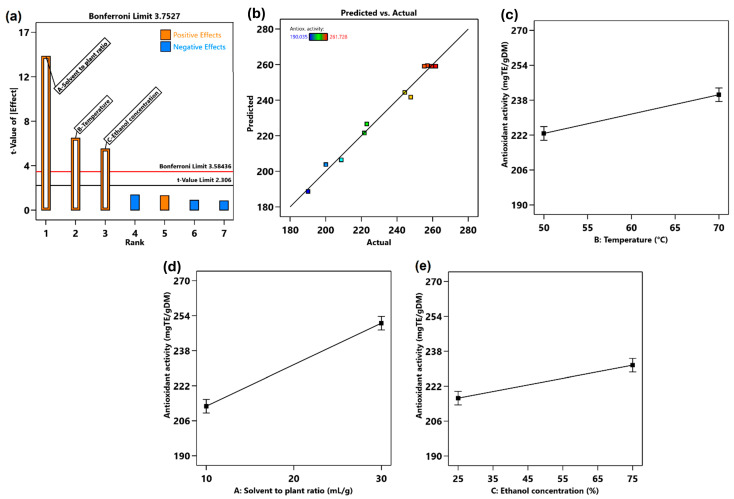
(**a**) Pareto chart for AA, (**b**) actual vs. predicted result, (**c**) effect of solvent-to-plant ratio, (**d**) temperature, and (**e**) ethanol concentration on AA.

The influence of MAE parameters on the maximum AA was studied (Figure 3). The effect and the interaction of the factors on the AA was shown through Pareto chart (Figure 3a) and a comparison of the predicted and actual results was conducted (Figure 3b). The relationship between AA and these variables was expressed using a reduced fitting model presented in Equation (2).

Y = 224.09 + 18.95 × A + 8.87 × B + 7.54 × C,(2)

where Y is the AA of hawthorn leaves and flowers extracts.

The model was significant (*p* < 0.0001), and it is clear that the AA was strongly affected by ethanol concentration, temperature, and solvent-to-plant ratio (Table 10 and Figure 3c–e). Pareto plot indicated that ethanol concentration was the greatest contributing factor in reaching the highest AA of extract from hawthorn leaves and flowers. One probable reason for this significance could be the solubility of antioxidant bioactive compounds in aqueous ethanol. The contributing factors on the AA of hawthorn extracts follow the order: ethanol concentration (39.2%) > temperature (8.6%) > solvent-to-plant ratio (6.2%). Temperature and solvent-to-plant ratio show a less significant effect on AA (*p* > 0.001, Table 10). During extraction at high temperatures, the compound stability may be affected as it is known that polyphenols are thermally labile [42]. Additionally, the interaction between the extraction parameters had no significant effect on increasing AA.

These results prove that the MAE process is an efficient and promising alternative to conventional methods, with the potential for further optimization to enhance efficiency. However, for successful large-scale implementation, energy consumption and cost-effectiveness must be carefully evaluated to maintain optimal extraction conditions. The full benefits of MAE will only be realized through continued efforts to scale up system designs, whether for large-batch or continuous processes. It is important to note that the initial capital investment and operating expenses for industrial microwave extraction systems can be high, necessitating a thorough cost-benefit analysis. Based on practical experience and microwave power requirements, a reasonable estimate of capital costs can be determined, serving as a guide for assessing the feasibility of scaling up this extraction process.

## 4. Conclusions

The purpose of this study was to improve the extraction efficiency of polyphenols with potential neuroprotective effects from hawthorn leaves and flowers using microwave technique. The influence of several parameters (extraction time, particle size of the vegetal material, temperature, ethanol concentration, and solvent-to-plant ratio) on the extraction of major polyphenols with neuroprotective effect (vitexin, hyperoside, rutin, and quercetin) found in hawthorn leaves and flowers was investigated. The results from the one-factor-at-a-time experimental design showed that the optimal extraction conditions of polyphenols from hawthorn leaves and flower were a 10 min extraction time, a particle size of the plant material smaller than 500 µm, a temperature of 60 °C, a 50% ethanol concentration, and a solvent-to-plant ratio of 20 mL/g. Under these conditions, the highest amounts of the interest polyphenols were achieved (10.40 ± 0.36 mg vitexin/g DM, 13.66 ± 0.32 mg hyperoside/g DM, 1.63 ± 0.21 mg rutin/g DM, 1.12 ± 0.09 mg quercetin/g DM, and TPC = 110.19 ± 1.68 mg GAE/g DM). MAE was compared with conventional extraction, yielding higher TPC and major component concentrations when using microwaves. A PCA biplot was performed to evaluate the correlations between the extraction conditions, TPC, and the main components from hawthorn extracts. A 2^3^ factorial design was used to estimate the significance of MAE parameters as solvent-to-plant ratio, temperature, and ethanol concentration in maximizing the TPC and AA of the extract obtained from hawthorn leaves and flowers. The achieved results indicated that the effects of extraction temperature, solvent-to-plant ratio, and ethanol concentration were significant (*p* < 0.05). Increasing these parameters led to the highest TPC (116.23 ± 2.85 mg GAE/g DM) and AA (237.6 ± 6.33 mg TE/g DM) in the extracts.

In summary, a one-factor-at-a-time design was used to identify extraction parameters for maximizing phytocompound content; however, its limitations necessitated the use of a 2^3^ full factorial design. This approach successfully optimized TPC and AA, while PCA uncovered correlations between extraction parameters, total phenolics, and key compounds in hawthorn leaves and flowers.

The resulting hawthorn extracts, enriched with polyphenols, especially those with neuroprotective properties, could be utilized in the development of dietary supplements aimed at promoting brain health and alleviating symptoms associated with neurodegenerative diseases.

## Figures and Tables

**Table 1:** Chemical composition and total phenolic content (TPC) of hawthorn extracts obtained via conventional and microwave extraction methods. The different letters (a,b) highlight the significant difference between groups (*p* < 0.05, ANOVA). Extraction conditions: ethanol concentration 50%, extraction time 10 min, temperature 60 °C, solvent-to-plant ratio 20 mL/g, stirring rate 900 rpm, particles size 160–500 μm. M1 and M2 are the method numbers. GAE stands for gallic acid equivalents and DM refers to dry hawthorn leaves and flowers.

Bioactive Compound	Retention Time, min	Bioactive Compounds Concentration, mg/g DM	
		Conventional (M1)	Microwave (M2)
Gallic acid	5.945	9.72 ± 0.99 ^a^	5.52 ± 0.39 ^b^
Chlorogenic acid	20.447	4.88 ± 0.30 ^a^	3.49 ± 0.28 ^b^
Vitexin	25.382	4.25 ± 0.10 ^b^	10.40 ± 0.36 ^a^
Rutin	32.889	0.21 ± 0.04 ^b^	1.63 ± 0.21 ^a^
Hyperoside	34.948	11.59 ± 0.04 ^b^	13.66 ± 0.32 ^a^
Isoquercetin	46.007	0.23 ± 0.03 ^b^	0.59 ± 0.12 ^a^
Quercetin	62.265	0.25 ± 0.05 ^b^	1.12 ± 0.09 ^a^
TPC, mg GAE/g DM		93.24 ± 3.83 ^b^	110.19 ± 1.68 ^a^

**Table 2:** Chemical composition and TPC of hawthorn extracts obtained at different extraction times. The different letters (a–c) highlight the significant difference between groups (*p* < 0.05, ANOVA). Extraction conditions: ethanol concentration 50%, temperature 60 °C, solvent-to-plant ratio 20 mL/g, stirring rate 900 rpm, particle sizes 160–500 μm.

Bioactive Compound	Retention Time, min	Bioactive Compounds Concentration, mg/g DM			
		Extraction Time			
		5 min	10 min	15 min	20 min
Gallic acid	5.945	5.18 ± 0.13 ^a^	5.52 ± 0.39 ^a^	5.43 ± 0.07 ^a^	4.54 ± 0.13 ^b^
Chlorogenic acid	20.447	2.03 ± 0.08 ^d^	3.49 ± 0.28 ^c^	5.07 ± 0.15 ^a^	4.54 ± 0.32 ^b^
Vitexin	25.382	8.06 ± 0.20 ^b^	10.40 ± 0.36 ^a^	6.89 ± 0.11 ^c^	7.84 ± 0.15 ^b^
Rutin	32.889	1.19 ± 0.17 ^b^	1.63 ± 0.21 ^a^	0.29 ± 0.04 ^c^	1.17 ± 0.06 ^b^
Hyperoside	34.948	12.49 ± 0.05 ^c^	13.66 ± 0.32 ^b^	15.17 ± 0.10 ^a^	13.89 ± 0.11 ^b^
Isoquercetin	46.007	0.42 ± 0.09 ^a,b^	0.59 ± 0.12 ^a^	0.38 ± 0.07 ^b^	0.47 ± 0.07 ^a,b^
Quercetin	62.265	0.89 ± 0.11 ^b^	1.12 ± 0.09 ^a^	0.86 ± 0.14 ^b^	0.87 ± 0.10 ^b^
TPC, mg GAE/g DM		101.31 ± 4.85 ^b^	110.19 ± 1.68 ^a^	110.30 ± 4.90 ^a^	109.66 ± 3.38 ^a^

**Table 3:** Chemical composition and TPC of hawthorn extracts obtained at different particle sizes of plant material. The different letters (a–c) highlight the significant difference between groups (*p* < 0.05, ANOVA). Extraction conditions: ethanol concentration 50%, extraction time 10 min, temperature 60 °C, solvent-to-plant ratio 20 mL/g, stirring rate 900 rpm. M2, M3, and M4 are the method numbers.

Bioactive Compound	Retention Time, min	Bioactive Compounds Concentration, mg/g DM		
		Particles Size		
		<160 μm (M3)	160–500 μm (M2)	500–1000 μm (M4)
Gallic acid	5.945	6.09 ± 0.08 ^b^	5.52 ± 0.39 ^c^	9.44 ± 0.15 ^a^
Chlorogenic acid	20.447	4.95 ± 0.18 ^a^	3.49 ± 0.28 ^c^	4.53 ± 0.12 ^b^
Vitexin	25.382	5.98 ± 0.38 ^b^	10.40 ± 0.36 ^a^	4.87 ± 0.11 ^c^
Rutin	32.889	0.54 ± 0.17 ^b^	1.63 ± 0.21 ^a^	0.18 ± 0.04 ^c^
Hyperoside	34.948	13.49 ± 0.76 ^a^	13.66 ± 0.32 ^a^	11.24 ± 0.21 ^b^
Isoquercetin	46.007	0.50 ± 0.05 ^a,b^	0.59 ± 0.12 ^a^	0.38 ± 0.07 ^b^
Quercetin	62.265	2.83 ± 0.15 ^a^	1.12 ± 0.09 ^b^	0.26 ± 0.04 ^c^
TPC, mg GAE/g DM		111.76 ± 4.04 ^a^	110.19 ± 1.68 ^a,b^	101.36 ± 6.71 ^b^

**Table 4:** Chemical composition and TPC of hawthorn extracts obtained at different extraction temperatures. The different letters (a–c) highlight the significant difference between groups (*p* < 0.05, ANOVA). Extraction conditions: ethanol concentration 50%, extraction time 10 min, solvent-to-plant ratio 20 mL/g, stirring rate 900 rpm, particles size 160–500 μm. M2, M5, and M6 are the method numbers.

Bioactive Compound	Retention Time, min	Bioactive Compounds Concentration, mg/g DM		
		Extraction Temperatures		
		50 °C (M5)	60 °C (M2)	70 °C (M6)
Gallic acid	5.945	7.09 ± 0.09 ^a^	5.52 ± 0.39 ^b^	6.98 ± 0.13 ^a^
Chlorogenic acid	20.447	5.44 ± 0.23 ^a^	3.49 ± 0.28 ^c^	4.94 ± 0.16 ^b^
Vitexin	25.382	5.61 ± 0.09 ^b^	10.40 ± 0.36 ^a^	5.37 ± 0.08 ^b^
Rutin	32.889	0.31 ± 0.04 ^b^	1.63 ± 0.21 ^a^	0.27 ± 0.05 ^b^
Hyperoside	34.948	13.57 ± 0.19 ^a^	13.66 ± 0.32 ^a^	12.79 ± 0.12 ^b^
Isoquercetin	46.007	0.33 0.03 ^b^	0.59 ± 0.12 ^a^	0.41 ± 0.05 ^b^
Quercetin	62.265	0.56 ± 0.07 ^b^	1.12 ± 0.09 ^a^	0.66 ± 0.17 ^b^
TPC, mg GAE/g DM		106.72 ± 2.70 ^b^	110.19 ± 1.68 ^a^	108.07 ± 2.41 ^a^

**Table 5:** Chemical composition and TPC of hawthorn extracts obtained at different ethanol concentrations. The different letters (a–d) highlight the significant difference between groups (*p* < 0.05, ANOVA). Extraction conditions: temperature 60 °C, extraction time 10 min, solvent-to-plant ratio 20 mL/g, stirring rate 900 rpm, particles size 160–500 μm. M2, M7, M8, M9, and M10 are the method numbers.

Bioactive Compound	Retention Time, min	Bioactive Compounds Concentration, mg/g DM				
		Ethanol Concentration				
		Water (M7)	25% (M8)	50% (M2)	75% (M9)	100% (M10)
Gallic acid	5.945	1.53 ± 0.19 ^d^	3.29 ± 0.25 ^b^	5.52 ± 0.39 ^a^	5.11 ± 0.22 ^a^	2.60 ± 0.44 ^c^
Chlorogenic acid	20.447	0.47 ± 0.06 ^d^	0.65 ± 0.23 ^c,d^	3.49 ± 0.28 ^a^	2.54 ± 0.38 ^b^	0.98 ± 0.19 ^c^
Vitexin	25.382	6.62 ± 0.36 ^d^	9.62 ± 0.25 ^b^	10.40 ± 0.36 ^a^	9.37 ± 0.18 ^b^	7.74 ± 0.47 ^c^
Rutin	32.889	0.79 ± 0.05 ^e^	1.22 ± 0.08 ^d^	1.63 ± 0.21 ^b^	2.26 ± 0.06 ^a^	1.41 ± 0.08 ^c^
Hyperoside	34.948	6.90 ± 0.32 ^d^	10.40 ± 0.25 ^c^	13.66 ± 0.32 ^b^	14.69 ± 0.28 ^a^	10.22 ± 0.40 ^c^
Isoquercetin	46.007	0.97 ± 0.10 ^b^	1.23 ± 0.08 ^a^	0.59 ± 0.12 ^c^	0.44 ± 0.13 ^c^	0.41 ± 0.02 ^c^
Quercetin	62.265	0.66 ± 0.05 ^c^	0.89 ± 0.19 ^b,c^	1.12 ± 0.09 ^b^	1.69 ± 0.21 ^a^	0.79 ± 0.12 ^c^
TPC, mg GAE/g DM		68.39 ± 6.33 ^d^	93.72 ± 3.22 ^b,c^	110.19 ± 1.68 ^a^	96.84 ± 6.69 ^b^	84.18 ± 6.54 ^c^

**Table 6:** Chemical composition and TPC of hawthorn extracts obtained at different solvent-to-plant ratio. The different letters (a–d) highlight the significant difference between groups (*p* < 0.05, ANOVA). Extraction conditions: ethanol concentration 50%, extraction time 10 min, temperature 60 °C, stirring rate 900 rpm, particles size 160–500 μm. M2, M11, M12, and M13 are the method numbers.

Bioactive Compound	Retention Time, min	Bioactive Compounds Concentration, mg/g DM			
		Solvent-to-Plant Ratio (S/P)			
		5 mL/g (M11)	10 mL/g (M12)	20 mL/g (M2)	30 mL/g (M13)
Gallic acid	5.945	2.34 ± 0.25 ^c^	3.06 ± 0.11 ^c^	5.52 ± 0.39 ^a^	4.56 ± 0.71 ^b^
Chlorogenic acid	20.447	0.99 ± 0.06 ^c^	1.28 ± 0.14 ^b,c^	3.49 ± 0.28 ^a^	1.32 ± 0.05 ^b^
Vitexin	25.382	6.49 ± 0.24 ^d^	8.05 ± 0.15 ^c^	10.40 ± 0.36 ^b^	11.14 ± 0.35 ^a^
Rutin	32.889	1.16 ± 0.05 ^b^	1.57 ± 0.24 ^a^	1.63 ± 0.21 ^a^	1.77 ± 0.04 ^a^
Hyperoside	34.948	6.37 ± 0.27 ^d^	8.35 ± 0.58 ^c^	13.66 ± 0.32 ^b^	15.27 ± 0.28 ^a^
Isoquercetin	46.007	0.16 ± 0.02 ^c^	0.29 ± 0.04 ^c^	0.59 ± 0.12 ^b^	0.78 ± 0.14 ^a^
Quercetin	62.265	0.48 ± 0.17 ^d^	0.75 ± 0.05 ^c^	1.12 ± 0.09 ^b^	1.42 ± 0.15 ^a^
TPC, mg GAE/g DM		93.72 ± 3.22 ^c^	101.19 ± 1.84 ^b^	110.19 ± 1.68 ^a^	111.80 ± 1.85 ^a^

**Table 7:** Factor loadings.

	PC1	PC2
Gallic acid	**0.911**	0.154
Chlorogenic acid	**0.948**	0.194
Vitexin	**−0.748**	0.578
Rutin	**−0.779**	0.400
Hyperoside	0.387	**0.865**
Isoquercetin	−0.505	0.327
Quercetin	−0.119	**0.723**
TPC	0.505	**0.669**

**Table 8:** Correlation matrix (Pearson (n)).

Variables	Gallic Acid	Chlorogenic Acid	Vitexin	Rutin	Hyperoside	Isoquercetin	Quercetin	TPC
Gallic acid	**1**	0.879	−0.523	−0.585	0.530	−0.383	−0.113	0.489
Chlorogenic acid	**0.879**	**1**	−0.589	−0.640	0.545	−0.450	0.076	0.523
Vitexin	−0.523	−0.589	**1**	0.874	0.262	0.523	0.343	−0.006
Rutin	−0.585	−0.640	0.874	**1**	0.118	0.207	0.326	−0.217
Hyperoside	0.530	0.545	0.262	0.118	**1**	0.011	0.479	0.686
Isoquercetin	−0.383	−0.450	0.523	0.207	0.011	**1**	0.192	0.096
Quercetin	−0.113	0.076	0.343	0.326	0.479	0.192	**1**	0.292
TPC	0.489	0.523	−0.006	−0.217	0.686	0.096	0.292	**1**

**Table 9:** Experimental design conditions, TPC and antioxidant activity (AA) of hawthorn extracts.

Runs	A: Solvent-to-Plant Ratio, mL/g	B: Temperature, °C	C: Ethanol Concentration, %	TPC, mg GAE/g DM	AA, mg TE/g DM
1	10 (–1)	50 (–1)	25 (–1)	56.42	190.04
2	30 (+1)	50 (–1)	25 (–1)	63.20	223.05
3	10 (–1)	70 (+1)	25 (–1)	68.84	208.72
4	30 (+1)	70 (+1)	25 (–1)	79.94	244.38
5	10 (–1)	50 (–1)	75 (+1)	81.39	200.09
6	30 (+1)	50 (–1)	75 (+1)	89.39	247.71
7	10 (–1)	70 (+1)	75 (+1)	88.47	221.69
8	30 (+1)	70 (+1)	75 (+1)	92.03	257.02
9	20 (0)	60 (0)	50 (0)	106.58	259.94
10	20 (0)	60 (0)	50 (0)	106.24	259.45
11	20 (0)	60 (0)	50 (0)	105.08	255.32
12	20 (0)	60 (0)	50 (0)	107.90	261.73

**Table 10:** Experimental design conditions, TPC and AA of hawthorn extracts. * *p* < 0.05; ** *p* < 0.01; *** *p* < 0.001.

(A) Source TPC	Sum of Squares	DF	Mean Square	F Value	*p*-Value	Significant
Model	1202.81	4	300.70	96.79	<0.0001	significant
A-Solvent-to-plant ratio	108.25	1	108.25	34.84	0.0011	**
B-Temperature	188.88	1	188.88	60.80	0.0002	***
C-Ethanol concentration	858.45	1	858.45	276.31	<0.0001	***
BC	47.23	1	47.23	15.20	0.0080	**
Residual	18.64	6	3.11			
Lack of Fit	14.59	3	4.86	3.60	0.1604	not significant
Pure Error	4.05	3	1.35			
Cor Total	3462.19	11				
R^2^	0.9847					
Adjusted R^2^	0.9746					
Predicted R^2^	0.9092					
Adeq Precision	40.3683					
C.V. %	2.02					
**(B) Source AA**	Sum of Squares	DF	Mean Square	F Value	*p*-value	Significant
Model	3957.74	3	1319.25	94.84	< 0.0001	significant
A-Solvent-to-plant ratio	2874.17	1	2874.17	206.62	< 0.0001	***
B-Temperature	628.75	1	628.75	45.20	0.0003	***
C-Ethanol concentration	454.82	1	454.82	32.70	0.0007	***
Residual	97.37	7	13.91			
Lack of Fit	75.37	4	18.84	2.57	0.2322	not significant
Pure Error	22.00	3	7.33			
Cor Total	7326.13	11				
R^2^	0.9760					
Adjusted R^2^	0.9657					
Predicted R^2^	0.9160					
Adeq Precision	29.3749					
C.V. %	1.58					

## Data Availability

Data are contained within the article.
